# Tumor Derived SIGLEC Family Genes May Play Roles in Tumor Genesis, Progression, and Immune Microenvironment Regulation

**DOI:** 10.3389/fonc.2020.586820

**Published:** 2020-11-09

**Authors:** Zheng Chen, Mincheng Yu, Lei Guo, Bo Zhang, Shuang Liu, Wentao Zhang, Binghai Zhou, Jiuliang Yan, Qianni Ma, Zhangfu Yang, Yongsheng Xiao, Yongfeng Xu, Hui Li, Qinghai Ye

**Affiliations:** Liver Cancer Institute, Zhongshan Hospital, Fudan University and Key Laboratory of Carcinogenesis and Cancer Invasion, Ministry of Education, Shanghai, China

**Keywords:** pan-cancer, tumor immune microenvironment, tumor genesis, tumor progression, bioinformatic analysis

## Abstract

**Background:**

SIGLEC family genes can also be expressed on tumor cells in different cancer types, and though it has been found that SIGLEC genes expressed by immune cells can be exploited by tumors to escape immune surveillance, functions of tumor derived SIGLEC expression in tumor microenvironment (TME) were barely investigated, which could play roles in cancer patients’ survival.

**Methods:**

Using bioinformatic analysis, mutation status of SIGLEC family genes was explored through the cBioPortal database, and expression of them in different tumors was explored through the UALCAN database. The GEPIA database was used to compare SIGLEC family genes’ mRNA between cancers and to generate a highly correlated gene list in tumors. A KM-plotter database was used to find the association between SIGLEC genes and survival of patients. The associations between SIGLEC family genes’ expression, immune infiltration, and immune regulators’ expression in TME were generated and examined by the TIMER 2.0 database; the differential fold changes of SIGLEC family genes in specific oncogenic mutation groups of different cancer types were also yielded by TIMER 2.0. The networks of SIGLEC family genes and highly correlated genes were constructed by the STRING database, and gene ontology and pathway annotation of SIGLEC family highly correlated genes were performed through the DAVID database.

**Results:**

SIGLEC family genes were highly mutated and amplified in melanoma, endometrial carcinoma, non-small cell lung cancer, bladder urothelial carcinoma, and esophagogastric adenocarcinoma, while deep deletion of SIGLEC family genes was common in diffuse glioma. Alteration of SIGLEC family genes demonstrated different levels in specific tumors, and oncogenic mutation in different cancer types could influence SIGLEC family genes’ expression. Most SIGLEC family genes were related to patients’ overall survival and progression free survival. Also, tumor derived SIGLEC family genes were related to tumor immune cell infiltration and may regulate TME by influencing chemokine axis.

**Conclusion:**

Our computational analysis showed SIGLEC family genes expressed by tumor cells were associated with tumor behaviors, and they may also influence TME through chemokine axis, playing vital roles in patients’ survival. Further experiments targeting tumor derived SIGLEC family genes are needed to confirm their influences on tumor growth, metastasis, and immune environment regulation.

## Introduction

SIGLEC family genes translate a group of proteins belonging to the immunoglobulin superfamily in mammal animals, and they can be divided into conservative (SIGLEC1, SIGLEC2, SIGLEC4, and SIGLEC15) and highly evolved (SIGLEC3, SIGLEC5, SIGLEC6, SIGLEC7, SIGLEC8, SIGLEC9, SIGLEC10, SIGLEC11, SIGLEC14, and SIGLEC16) teams, which are widely expressed on immune cell populations’ membrane, mainly involving endocytosis and immune regulation in various diseases (1–5). SIGLEC family genes’ protein on immune cells could bind to sialylated oligosaccharides, a type of glycoprotein, expressed by self or non-self cells, which in turn activate or inhibit immune cell function themselves or by binding to other functional kinase protein, providing targets for immune therapy (6).

It has been found that tumor cells can express sialylated ligands for SIGLEC receptors on immune cells, depressing immune cell function to escape immune surveillance, such as SIGLEC7 and SIGLEC9 on natural killer cell (7–12). However, SIGLEC family genes can also be expressed by tumor cells across cancer types, and recently, studies have found SIGLEC15 expressed by tumor cells or macrophages in mouse melanoma model could directly depress CD8+ T cell infiltration and function in tumor microenvironment through binding to presumptive target on CD8+ T cells (13–15). The explicit roles of tumor intrinsic SIGLEC family genes’ expression on patients’ survival, disease progression, and immune regulation in tumor microenvironment were still unknown, and we used bioinformatic analysis to find whether tumor derived expression of SIGLEC family genes played roles in those aspects, which could provide new thoughts for cancer immune therapy.

## Materials and Methods

### Mutation and Alteration Frequency of SIGLEC Family Genes

**Figure 1:**
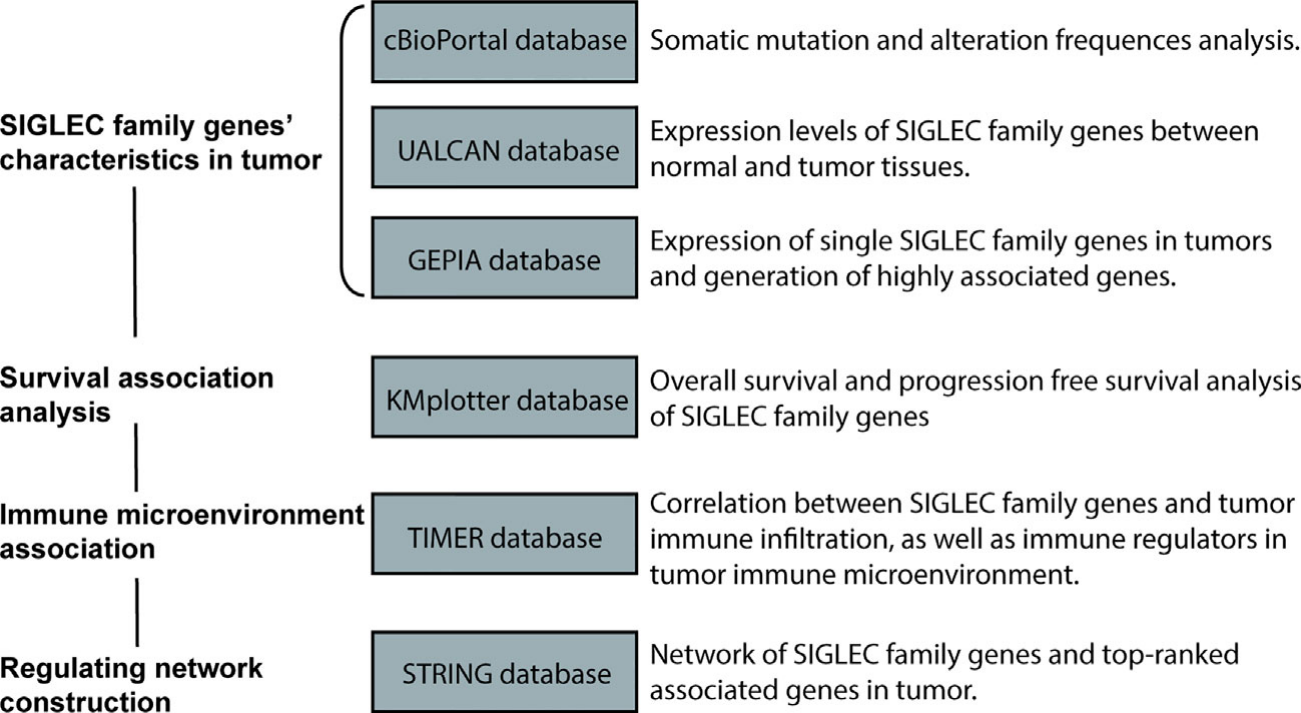
Workflow of this study.

cBioPortal database (https://www.cbioportal.org) was used to explore the mutation frequency of SIGLEC family genes in 33 types of tumors (melanoma, endometrial carcinoma, esophagogastric adenocarcinoma, non-small cell lung cancer, colorectal adenocarcinoma, ovarian epithelial tumor, cervical squamous cell carcinoma, bladder urothelial carcinoma, esophageal squamous cell carcinoma, sarcoma, head and neck squamous cell carcinoma, pancreatic adenocarcinoma, hepatocellular carcinoma, leukemia, prostate adenocarcinoma, invasive breast carcinoma, ocular melanoma, diffuse glioma, non-seminomatous germ cell tumor, renal non-clear cell carcinoma, pleural mesothelioma, adrenocortical carcinoma, glioblastoma, renal clear cell carcinoma, cervical adenocarcinoma, cholangiocarcinoma, mature B-cell neoplasm, pheochromocytoma, miscellaneous neuroepithelial tumor, undifferentiated stomach adenocarcinoma, seminoma, well-differentiated thyroid cancer, thymic epithelial tumor), which is an integrative database for analysis of mutation in various cancer types, containing somatic mutation and copy number variation data from the cancer genome atlas (TCGA) database (https://www.cancer.gov/about-nci/organization/ccg/research/structural-genomics/tcga) and other published articles (16, 17). The data of each tumor in TCGA Pan-Cancer projects were selected for analysis and demonstration. The data of alteration frequency (amplification, deletion, mutation, fusion, log2 transformed expression>=2 or log2 transformed expression<=-2) of each SIGLEC family gene were also generated and downloaded from the official website for further analysis (**Figure 1**).

### mRNA Expression Levels of SIGLEC Family Genes and Comparison Between Normal and Tumor Tissues

mRNA expression levels of SIGLEC family genes were compared between normal and tumor tissues of each cancer type, using the UALCAN database (http://ualcan.path.uab.edu), which is an official website for comprehensive analysis of cancer data from the TCGA database (18). The corresponding significance of examination in each comparison test was generated by website, and the results were marked with asterisks for illustration.

### Influence of SIELCE Family Gene mRNA Expression on Patients’ Overall Survival and Progression Free Survival

The KM-plotter database (http://www.kmplot.com/analysis/index.php?p=service) was used to analyze the association between SIGLEC family genes’ mRNA expression and patients’ overall survival (OS) and progression free survival (PFS). KM-plotter database is an portal website for analysis of association between gene expression and patients survival, using data from previous performed analysis, such as data from TCGA Pan-cancer projects and GEO database, covering micro RNA, long non-coding RNA, mRNA, and epigenetic information (19). The hazard ratio, 95% confidence interval, and *p* value of each analysis were generated by website, and in this analysis, best cutoff *p* values were deployed to subgroup patients into high- and low-expression groups.

### mRNA Expression of SIGLEC Family Genes in Single Tumor and the List of SIGLEC Family Highly Correlated Genes

The Gene Expression Profiling Interactive Analysis (GEPIA) database (http://gepia.cancer-pku.cn) is a comprehensive database for tumor gene expression analysis, providing a portal for analyzing specific genes in 32 types of cancer, using data from TCGA Pan-Cancer project and GTEx database (https://www.gtexportal.org) (20, 21). In this analysis, median expression of SIGLEC family genes (transcript per million base, TPM) in each tumor type were downloaded from the website for demonstration, and the first 50 highly correlated genes of each SIGLEC family gene in tumor were generated through the website and were combined as SIGLEC family highly correlated genes for further analysis.

### Correlation Between SIGLEC Family Genes, Immune Infiltration, and Immune Regulators

TIMER 2.0 (http://cistrome.shinyapps.io/timer) is a web-derived tool for analysis of tumor immune infiltration, which provides scores of 6 types of infiltrating immune cells (B cell, CD4+ T cell, CD8+ T cell, myeloid-derived dendritic cell, macrophages, and neutrophils) in tumors (22, 23). The correlation between SIGLEC family genes and six types of infiltrating immune cell were examined in each of 32 cancers, the data of which were from the TCGA database, and the results of examination were downloaded for demonstration. The correlation between SIGLEC family genes and well-known immune regulators were also analyzed through TIMER 2.0 for demonstration. TIMER 2.0 can also analyze differentially expressed genes between specific oncogene mutation groups, and SIGLEC family genes were input for analysis of well-known oncogenic mutation in specific tumors.

### Network of SIGLEC Family Genes and Highly Correlated Genes in Tumors

The networks of SIGLEC family genes and highly correlated genes were yielded by STRING database (https://string-db.org), which can construct network of selective genes given results of formerly examined correlation in articles (24). The results were downloaded from the website, and Cytoscape (version: 3.7.1) was used to illustrate the correlation (25). The cytoHubba tool in Cytoscape was used to procure the core network and the top-10 leading node genes (26).

### Gene Ontology of SIGLEC Family Highly Correlated Genes

The DAVID database (https://david.ncifcrf.gov) was used for gene ontology annotation of SIGLEC family highly correlated genes, which is a useful web tool for functional annotation (biological process, cellular compartment, and molecular function) of gene lists, and is also a tool for gene symbol transformation (27). It can link to the KEGG database (https://www.kegg.jp) for pathway annotation (28).

### Statistics

All statistical examinations were performed by database derived tools, and *p* value under 0.05 was considered significant. All heat maps in this analysis were constructed in R environment (version: 3.6.1), using R studio (version: 1.2.1335) and pheatmap (29–31). R package of graphics was used to construct a forrest graph of hazard ratio in survival analysis, and ggplot2, topGO, and clusterProfiler were used to generate the gene ontology graph (32–34).

## Results

### Alteration Frequency of SIGLEC Family Genes Across Different Cancer Types

**Figure 2:**
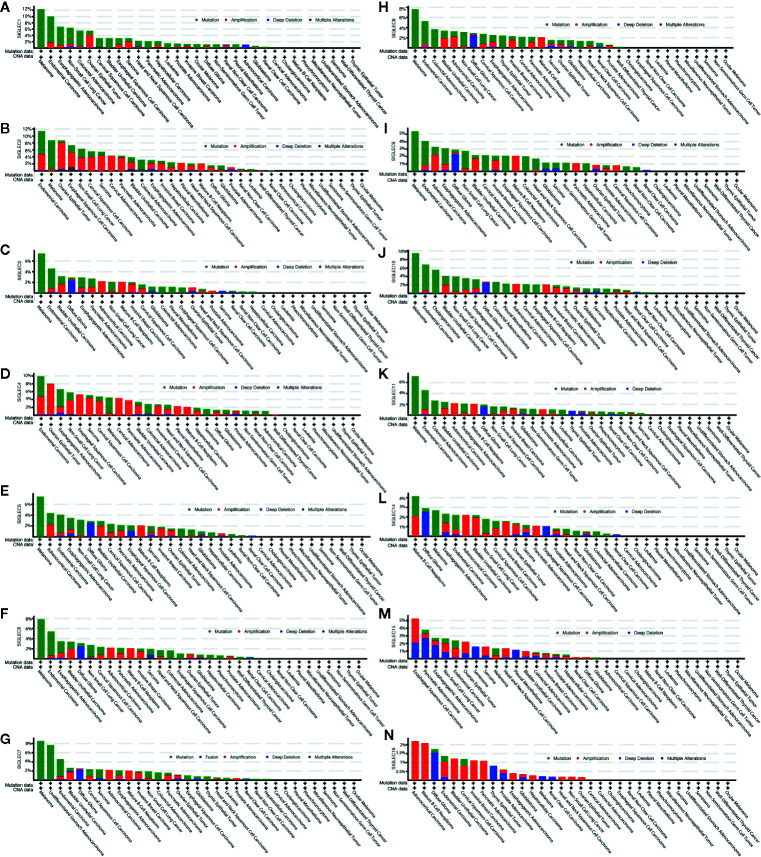
Mutation frequency of SIGLEC family genes across different cancer types in cBioPortal database. **(A)**. Mutation frequency of SIGLEC1 across cancers. **(B)**. Mutation frequency of SIGLEC2 across cancers. **(C)**. Mutation frequency of SIGLEC3 across cancers. **(D)**. Mutation frequency of SIGLEC4 across cancers. **(E)**. Mutation frequency of SIGLEC5 across cancers. **(F)**. Mutation frequency of SIGLEC6 across cancers. **(G)**. Mutation frequency of SIGLEC7 across cancers. **(H)**. Mutation frequency of SIGLEC8 across cancers. **(I)**. Mutation frequency of SIGLEC9 across cancers. **(J)**. Mutation frequency of SIGLEC10 across cancers. **(K)**. Mutation frequency of SIGLEC11 across cancers. **(L)**. Mutation frequency of SIGLEC14 across cancers. **(M)**. Mutation frequency of SIGLEC15 across cancers. **(N)**. Mutation frequency of SIGLEC16 across cancers.

SIGLEC family genes (SIGLEC1 or CD169, SIGLEC2 or CD22, SIGLEC3 or CD33, SIGLEC4 or MAG, SIGLEC5, SIGLEC6, SIGLEC7, SIGLEC8, SIGLEC9, SIGLEC10, SIGLEC11, SIGLEC14, SIGLEC15, SIGLEC16) were also expressed in tumor cells, and we used the cBioPortal database to find mutation status of SIGLEC family genes in different cancer types. Results showed mutation frequencies of SIGLEC1, SIGLEC2, and SIGLEC10 were relatively high among all SIGLEC family genes, and SIGLEC family genes were highly mutated in melanoma (except SIGLEC15 and SIGLEC16). However, in ocular melanoma, miscellaneous neuroepithelial tumor, seminoma, cholangiocarcinoma, undifferentiated stomach adenocarcinoma, pheochromocytoma, well-differentiated thyroid cancer, non-seminomatous germ cell tumor, cervical adenocarcinoma, and pheochromocytoma mutation of SIGLEC family genes were rare. Of notice, while most SIGLEC family genes rarely mutated in cholangiocarcinoma and undifferentiated stomach adenocarcinoma, SIGLEC10 and SIGLEC7 were respectively highly mutated in each of them. Alteration frequencies (mutation, amplification, deep deletion, and multiple mutation) of SIGLEC family genes were also high in endometrial carcinoma, non-small cell lung cancer, bladder urothelial carcinoma, and esophagogastric adenocarcinoma. For endometrial carcinoma, non-small cell lung cancer and bladder urothelial carcinoma, mutation, and amplification of SIGLEC family genes were both high; for esophagogastric adenocarcinoma, mutation, amplification, and deep deletion of SIGLEC family genes were all common. Also, in diffuse glioma, deep deletion of SIGLEC family genes was common in comparison to 32 other types of tumor, and SIGLEC15 seemed to be deeply deleted in various tumors, while SIGLEC16 was amplified in mutating cancer types (**Figure 2**).

### Expression Levels of SIGLEC Family Genes Between Normal and Tumor Tissues Across Cancer Types

**Figure 3:**
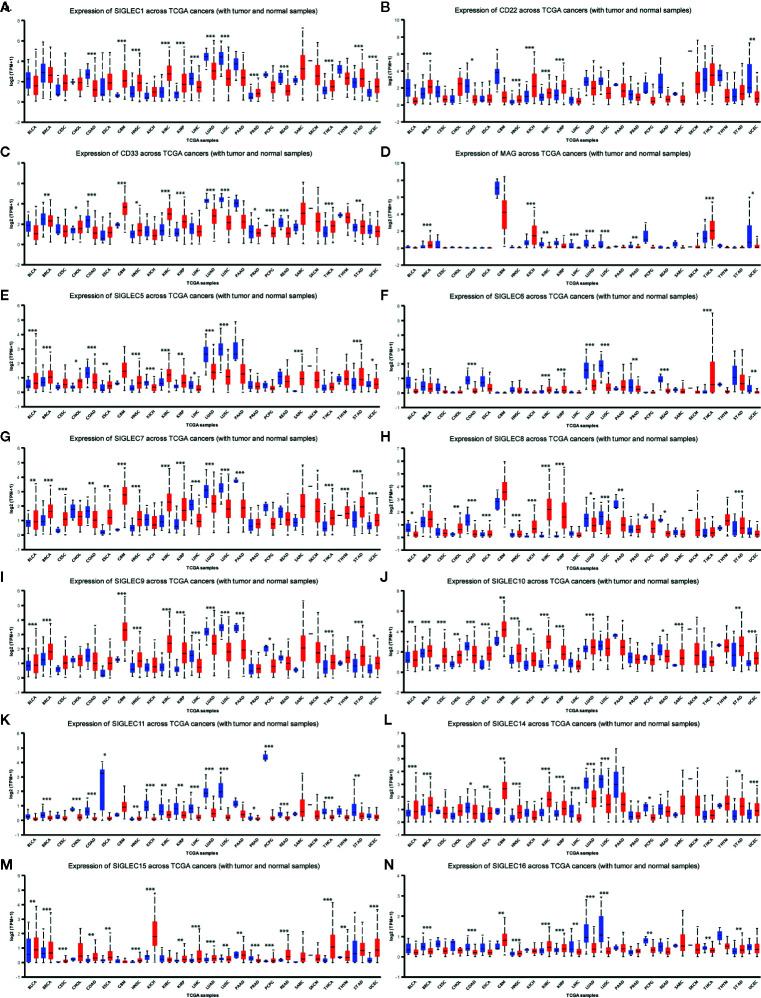
SIGLEC family genes were differentially expressed between different tumor and corresponding normal tissues. **(A)**. Expression of SIGLEC1 in normal and cancer tissues of TCGA database. **(B)**. Expression of CD22 (SIGLEC2) in normal and cancer tissues of TCGA database. **(C)**. Expression of CD33 (SIGLEC3) in normal and cancer tissues of TCGA database. **(D)**. Expression of MAG (SIGLEC4) in normal and cancer tissues of TCGA database. **(E)**. Expression of SIGLEC5 in normal and cancer tissues of TCGA database. **(F)**. Expression of SIGLEC6 in normal and cancer tissues of TCGA database. **(G)**. Expression of SIGLEC7 in normal and cancer tissues of TCGA database. **(H)**. Expression of SIGLEC8 in normal and cancer tissues of TCGA database. **(I)**. Expression of SIGLEC9 in normal and cancer tissues of TCGA database. **(J)**. Expression of SIGLEC10 in normal and cancer tissues of TCGA database. **(K)**. Expression of SIGLEC11 in normal and cancer tissues of TCGA database. **(L)** Expression of SIGLEC14 in normal and cancer tissues of TCGA database. **(M)**. Expression of SIGLEC15 in normal and cancer tissues of TCGA database. **(N)**. Expression of SIGLEC16 in normal and cancer tissues of TCGA database. (*p < 0.05, **p < 0.01, ***p < 0.001; Bladder Urothelial Carcinoma, BLCA, normal=19, tumor=408; Breast invasive carcinoma, BRCA, normal=114, tumor=1097; Cervical squamous cell carcinoma and endocervical adenocarcinoma, CESC, normal=3, tumor=305; Cholangiocarcinoma, CHOL, normal=9, tumor=36; Colon adenocarcinoma, COAD, normal=41, tumor=286; Esophageal carcinoma, ESCA, normal=11, tumor=184; Glioblastoma multiforme, GBM, normal=5, tumor=156; Head and Neck squamous cell carcinoma, HNSC, normal=44, tumor=520; Kidney Chromophobe, KICH, normal=25, tumor=67; Kidney renal clear cell carcinoma, KIRC, normal=72, tumor=533; Kidney renal papillary cell carcinoma, KIRP, normal=32, tumor=290; Liver hepatocellular carcinoma, LIHC, normal=50, tumor=371; Lung adenocarcinoma, LUAD, normal=59, tumor=515; Lung squamous cell carcinoma, LUSC, normal=52, tumor=503; Pancreatic adenocarcinoma, PAAD, normal=4, tumor=178; Prostate adenocarcinoma, PRAD, normal=52, tumor=497; Pheochromocytoma and Paraganglioma, PCPG, normal=3, tumor=179; Rectum adenocarcinoma, READ, normal=10, tumor=166; Sarcoma, SARC, normal=2, tumor=260; Skin Cutaneous Melanoma, SKCM, normal=1, tumor=472; Thyroid carcinoma, THCA, normal=59, tumor=505; Thymoma, THYM, normal=2, tumor=120; Stomach adenocarcinoma, STAD, normal=34, tumor=415; Uterine Corpus Endometrial Carcinoma, UCEC, normal=35, tumor=546. Blue boxplot: normal expression; red boxplot: tumor expression.)

We wondered whether SIGLEC family genes were differentially expressed between normal and tumor tissues in various cancer types, and we used the UALCAN database to examine their expression levels. We found mRNA expression levels of SIGLEC family genes were different between normal and tumors tissues, and extremely in breast invasive carcinoma (BRCA), colon adenocarcinoma (COAD), head and neck squamous cell carcinoma (HNSC), kidney renal clear cell carcinoma (KIRC), kidney renal papillary cell carcinoma (KIRP), liver hepatocellular carcinoma (LIHC), lung adenocarcinoma (LUAD), lung squamous cell carcinoma (LUSC), thyroid carcinoma (THCA), stomach adenocarcinoma (STAD), and uterine Corpus Endometrial Carcinoma (UCEC), almost all expressional difference of SIGLEC family genes achieved significance. In glioblastoma multiform (GBM), HNSC, KIRC, KIRP, THCA, and STAD, most SIGLEC family genes were highly expressed in tumor samples, while in COAD, LIHC, LUAD, and LUSC, most SIGLEC family genes were down-regulated in tumors. Also, SIGLEC11 mRNA expression was down-regulated in 19 out of 24 cancer types (except GBM, HNSC, THCA, and sarcoma [SARC], prostate adenocarcinoma [PRAD]), and SIGLEC15 was up-regulated in 18 out of 24 cancer types (except BRCA, KIRC, LUSC, PRAD, thymoma [THYM], and skin cutaneous melanoma [SKCM]) (**Figure 3**).

### mRNA Expression of SIGLEC Family Genes Differed in Different Tumors, and Oncogenic Mutation Changes of Different Tumors Were Related to SIGLEC Family Genes

**Figure 4:**
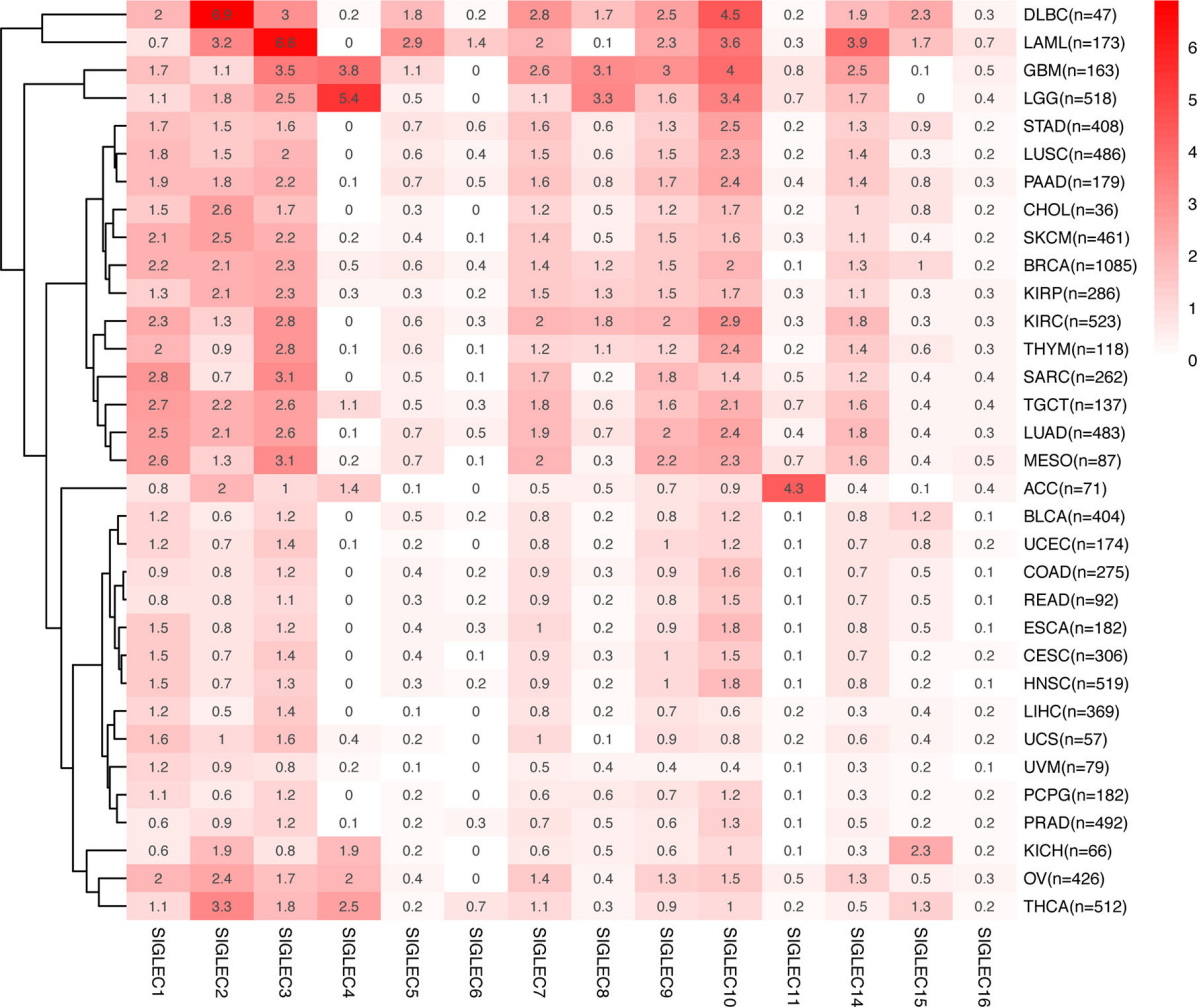
mRNA Expression of SIGLEC family genes differed in various tumors. Average mRNA expression (TPM) of SIGLEC family genes in different tumors from GEPIA database.

**Figure 5:**
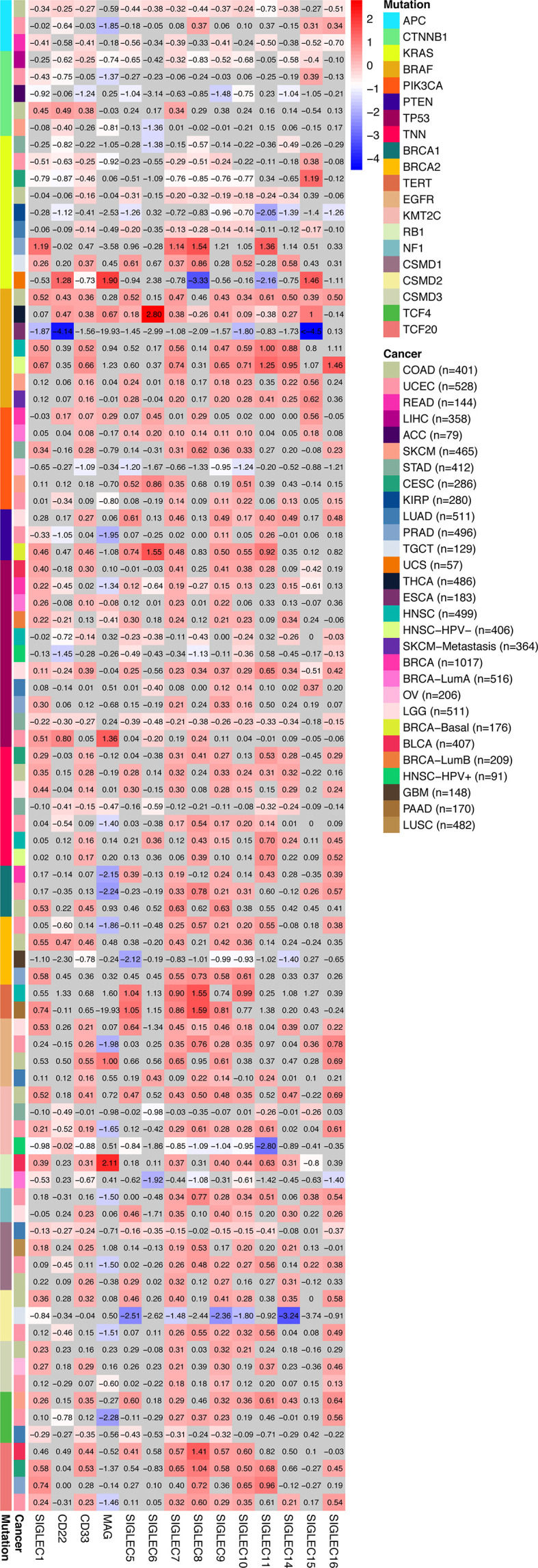
Tumor specific oncogenic gene mutation was related to SIGLEC mRNA expression in tumors of different origin. Fold changes of log2-transformed differential expression of SIGLEC family genes in specific oncogene mutation groups of different tumors (calculated by TIMER 2.0 database). Fold changes with significance were marked with bright colors, and non-significant fold changes were marked with grey background. (*p*<0.05 was considered significant.)

We compared the median TPM expression of SIGLEC family genes between cancer types (from GEPIA database), and results showed some SIGLEC family genes were highly expressed in specific tumors (**Figure 4**). Using TIMER 2.0 database, we found tumor specific oncogenic mutation could influence expression levels of SIGLEC family genes. In APC mutation groups of COAD and rectum adenocarcinoma (READ), most SIGLEC family genes were significantly down-regulated; in the CTNNB1 mutation group of LIHC, all SIGLEC family genes were down-regulated (10 out of 14 achieved significance). In other common mutations of tumors, such as PTEN, TP53, KRAS, and PIK3CA, SIGLEC family genes also demonstrated significant expressional changes in mutation groups of different cancer types (**Figure 5**).

### Tumor Derived mRNA Expression of SIGLEC Family Genes Were Highly Correlated to Patients’ Overall Survival and Progression Survival Across Cancer Types

**Figure 6:**
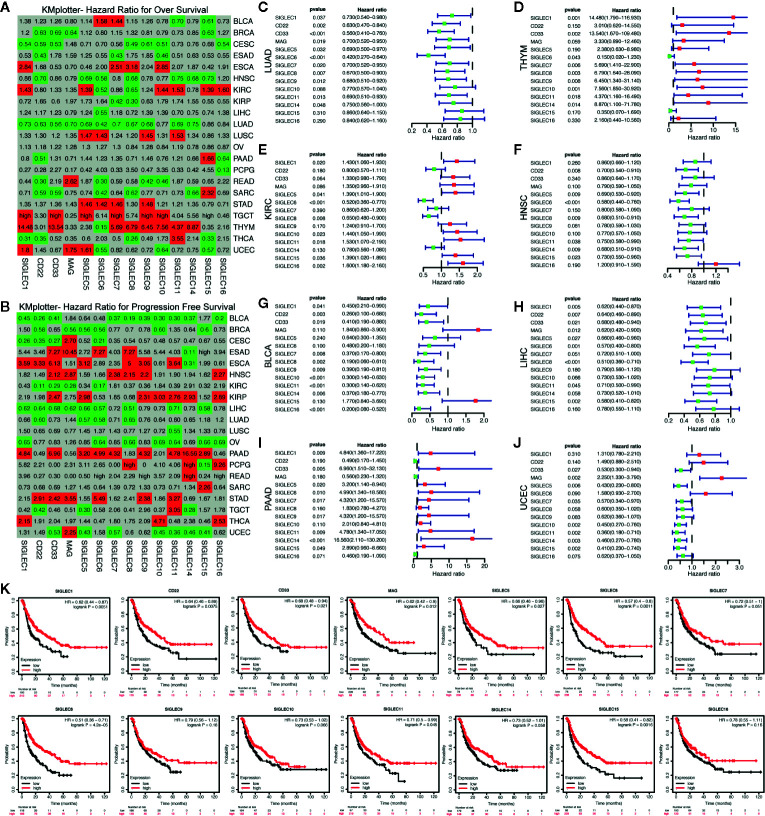
mRNA expression levels of SIGLEC family genes in different tumors were related to patients’ overall survival and progression free survival. **(A)**. Hazard ratio (HR) for patients’ overall survival (OS) of different tumors (calculated by KMplotter database). **(B)**. Hazard ratio (HR) for patients’ progression free survival (PFS) of different tumors (calculated by KMplotter database). (HRs less than 1 with significance were marked with green background as protective factors, and HRs over 1 were marked with red background as risk factors. Non-significant HRs were marked with grey background. HRs over 50 were replaced with “high.”) **(C–F)**. HRs, 95% confidence interval and *p* value for SIGLEC family genes in association with patients’ OS in LUAD, THYM, KIRC, and HNSC. **(G–J)**. HRs, 95% confidence interval and *p* value for SIGLEC family genes in association with patients’ PFS in BLCA, LIHC, PAAD, and UCEC. (Green dots represented protective factors, while red dots represented risk factors.) **(K)**. Survival curves for SIGLEC family genes in association with patients’ PFS in LIHC.

We associated mRNA expression of SIGELC family genes with patients’ survival, using the KM-plotter database, and found SIGLEC family genes were related to patients’ overall survival (OS) and progression free survival (PFS) in most cancer types (**Figures 6A, B**). Concerning OS of patients with various tumors, most SIGLEC family genes in LUAD, THYM, KIRC, and HNSC showed significant correlation, and in HNSC and LUAD, most SIGLEC family genes showed protective roles, while in THYM and KIRC, most SIGLEC family genes were risk factors (**Figures 6C–F**). In LIHC and pancreatic adenocarcinoma (PAAD), only one (SIGLEC6) and three (SIGLEC2, SIGLEC15, and SIGLEC16) SIGLEC family genes were related to patients’ OS, respectively; however, when it comes to PFS, most SIGELC family genes changed to protective and risk factors in LIHC and PAAD, correspondingly. Also, in bladder urothelial carcinoma (BLCA) and UCEC, most SIGLEC family genes were significant protecting factors for PFS, and only MAG (SIGLEC4) was a risk factor for PFS in UCEC (**Figures 6G–K**).

### Tumor Intrinsic mRNA Levels of SIGLEC Family Genes Were Closely Related to Tumor Immune Cell Infiltration

**Figure 7:**
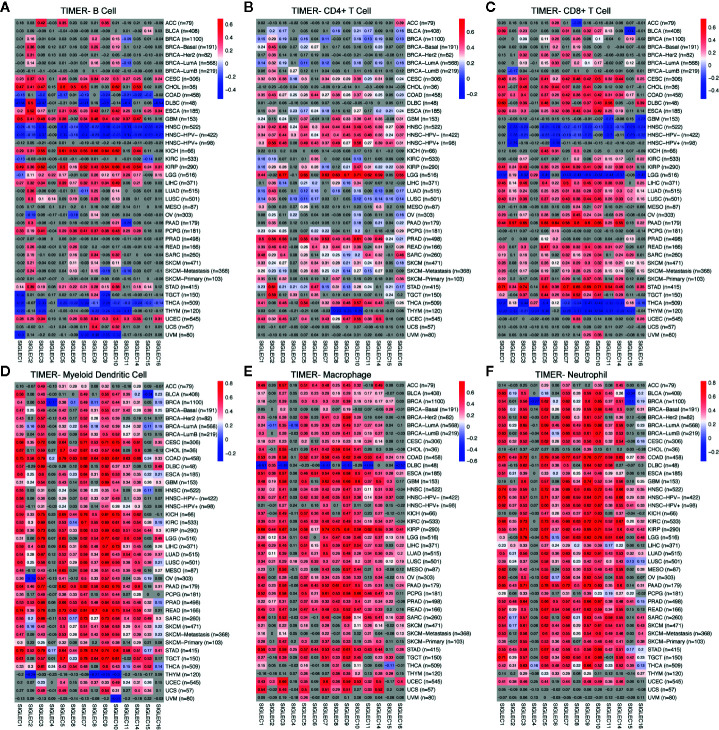
Coefficients for correlation between SIGLEC family genes and immune cell infiltration of different tumors in TIMER database. **(A)**. Coefficients for correlation between B cell infiltration score and SIGLEC family genes in different tumors. **(B)**. Coefficients for correlation between CD4+ T cell infiltration score and SIGLEC family genes in different tumors. **(C)**. Coefficients for correlation between CD8+ T cell infiltration score and SIGLEC family genes in different tumors. **(D)**. Coefficients for correlation between myeloid dendritic cell infiltration score and SIGLEC family genes in different tumors. **(E)**. Coefficients for correlation between macrophage infiltration score and SIGLEC family genes in different tumors. **(F)**. Coefficients for correlation between neutrophil infiltration score and SIGLEC family genes in different tumors. (Coefficients with significance were marked with bright colors, while non-significant coefficients were marked with grey background. *P* value under 0.05 was considered significant. The association was generated with tumor purification adjusted.)

Former studies showed SIGLEC family genes were expressed on immune cells and were involved in immune cell activation and adaptation; also, recently, SIGLEC15 was found to be able to dampen CD8+ T cell infiltration and function in mouse melanoma model. We wondered whether tumor intrinsic expression of SIGLEC family genes could influence immune infiltration in a tumor microenvironment. Using TIMER 2.0 to examine the correlation between SIGLEC family genes and six types of infiltrating immune cells (B cell, CD4+ T cell, CD8+ T cell, myeloid dendritic cell, macrophage, and neutrophil), we found in specific tumors, such as HNSC, THCA, and THYM, SIGLEC family genes were negatively correlated with B cell and CD8+ T cell infiltration, while in cervical squamous cell carcinoma and endocervical adenocarcinoma (CESC), esophageal carcinoma (ESCA), and KIRP, most SIGLEC family genes were positively correlated with B cell and CD8+ T cell (**Figures 7A–C**). Also, it turned out that SIGLEC family genes were highly correlated with dendritic cell, macrophage, and neutrophil in various cancer types (**Figures 7D–F**). Of notice, in brain lower grade glioma (LGG), most SIGLEC family genes were highly correlated with CD4+ T cell and negatively correlated with CD8+ T cell infiltration; in LIHC, LUAD, LUSC, PAAD, READ, SARC, SKCM, and stomach adenocarcinoma (STAD), most SIGLEC family genes were positively correlated with CD8+ T cells. Besides, in comparison to READ, COAD tissues additionally showed negative correlation between SIGLEC family genes and B cell infiltration.

### SIGLEC Family Genes Expressed in Tumor Cells Could Influence Immune Regulators in Tumor Microenvironment, Such as Chemokine Axis, Immune Stimulator, Immune Inhibitor, and MHC Molecular

**Figure 8:**
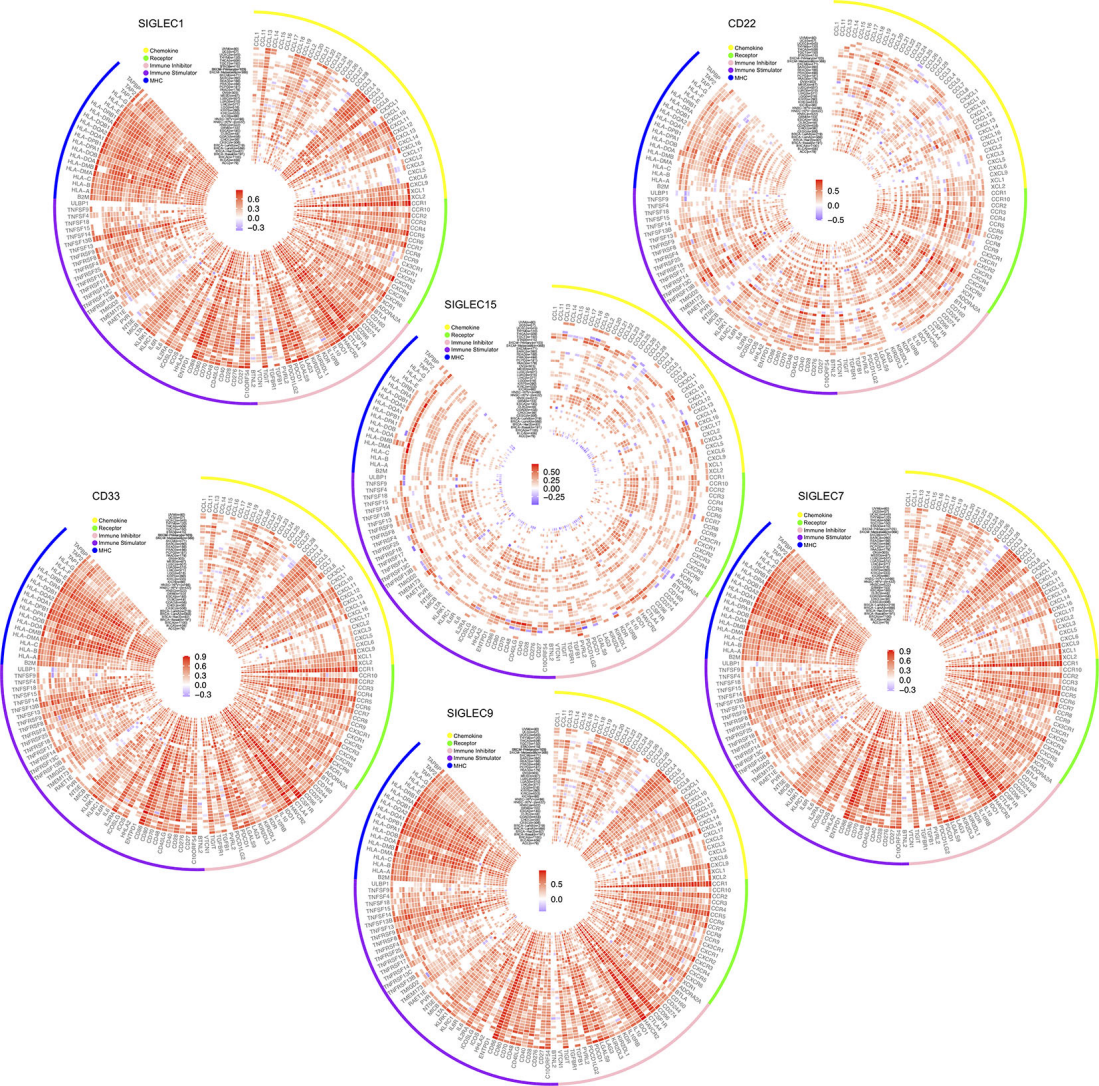
Correlation between mRNA expression of SIGLEC family genes (SIGLEC1, CD22 & SIGLEC2, CD33 & SIGLEC3, SIGLEC7, SIGLEC9, SIGLEC15) and immune regulators. (Coefficients with significance, calculated by TIMER 2.0 database, were shown with gradient color changes, and non-significant coefficients were replaced by blank space. *P* value under 0.05 was considered significant. The association was generated with tumor purification adjusted.)

After finding SIGLEC family genes expressed by tumor cells of various cancer types were closely related to tumor immune infiltration, we further examined the correlation between SIGLEC family genes and immune regulators in tumor. Since former studies have found SIGLEC1 (CD169), SIGLEC2 (CD22), SIGLEC3 (CD33), SIGLEC7, SIGLEC9, and SIGLEC15 expressed by immune cell populations could be manipulated by tumor cells to escape immune surveillance, we focused on the five SIGLEC family members. We found SIGLEC family genes were significantly correlated to a wide spectrum of immune regulators, including chemokine axis, immune stimulators, inhibitors, and MHC molecular, such as CD28, CD40, CD40LG, ICOS, LAG3, PDCD1, CD274, and CTLA4. Though some SIGLEC genes showed non-significant correlation with immune regulators, most coefficients were significant with absolute value over 0.5, indicating their active roles of immune regulation in tumor microenvironment (**Figure 8**).

**Figure 9:**
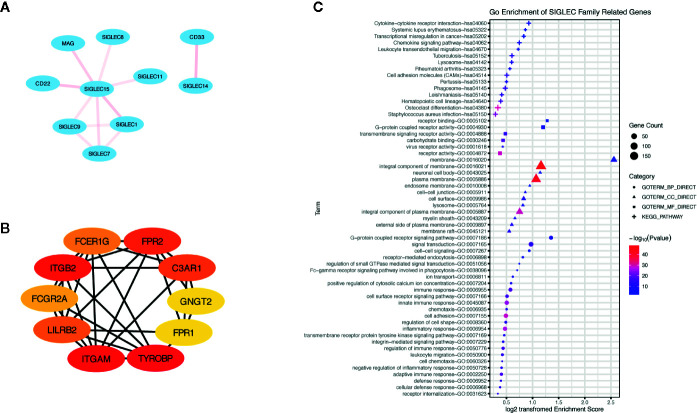
Genes highly correlated with SIGLEC family in tumors were enriched in immune regulation of tumor microenvironment. **(A)** Correlation between SIGLEC family genes. The correlation was calculated by STRING database, and transparency of edges between different genes reflected combined score between two-linked genes. **(B)** Highly SIGLEC family correlated genes (provided by GEPIA database) were calculated by STRING database, and top 10 connecting genes were selected by CytoHuba software. (The rank of genes was demonstrated by color changes, and the first ranked gene was marked with red bubble.) **(C)**. Gene ontology of highly SIGLEC family correlated genes. (Terms with gene count over 6 were shown, and *p* value under 0.05 was considered significant.)

Additionally, we generated a list of SIGLEC family highly correlated genes in tumor through GEPIA database, and we constructed a network of them through the STRING database (**Figures 9A–B**). The top ranked 10 node genes were TYROBP, ITGAM, ITGB2, FPR2, C3AR1, LILRB2, FCER1G, FCGR2A, GNGT2, and FPR1. Further gene ontology and pathway enrichment of SIGLEC family highly correlated genes, through DAVID database, showed those genes were most immune function related and enriched in cytokine-cytokine receptor interaction, chemokine signaling pathway, and leukocyte transendothelial migration (**Figure 9C**).

## Discussion

In our analysis, the alteration status of SIGLEC family genes differed in different cancer types. In melanoma, endometrial carcinoma, non-small cell lung cancer, bladder urothelial carcinoma, and esophagogastric adenocarcinoma, mutation and amplification of SIGLEC family genes were high, while deep deletion was commonly seen in diffuse glioma. We believed that in epithelium derived tumors, high proliferation status of tumor tissue may cause high frequencies of mutation and amplification of SIGLEC family genes in tumor cells; however, in diffuse glioma, the tumor microenvironment was different from other cancer types, which may redirect the adaptation of tumor cells. SIGLEC family genes were further associated with specific oncogene mutation in different cancer types and were differentially expressed between patients of mutation and non-mutation groups, such as APC in COAD and READ, or CTNNB1 in LIHC. Those mutations were formerly found to be related to degrees of tumor malignant behaviors or carcinogenesis, and we thought evolution of tumor cells may somehow drive expression changes of SIGLEC family genes.

SIGLEC family genes were highly correlated with patients’ OS and PFS across cancer types. Especially in LUAD, THYM, KIRC, and HNSC, most SIGLEC family genes were related to OS with significance, while in LIHC and PAAD, most of them were involved in PFS of patients. The hazard ratio for SIGLEC family genes differed in different cancer types: most SIGLEC family genes showed protective roles in some cancer types, while they demonstrated risking roles in the other. Also, though the hazard ratios for OS and PFS were consistent for most SIGLEC genes, in a few tumors, some SIGLEC family members were both risk factors and protective factors for OS and PFS. We thought expression levels of SIGLEC family genes in different cancer types may influence tumor malignant traits through different mechanisms, and their involvement in a special tumor microenvironment of different cancer types may also cause the survival difference across cancer types.

Former studies of SIGLEC family genes in tumors were mainly about their functional roles on immune cell populations, facilitating tumor growth, and immune escape in different cancer types, such as inhibitory SIGLEC9 and SIGLEC7 expressed on natural killer cells, or SIGLEC6 expressed on mast cells in colorectal cancer, which could be exploited by tumor cells through increased sialylation and glycosylation (7, 35–41). A recent study concerning SIGLEC15 also showed expression of SIGLEC15 by macrophages or tumor cells could directly depress function of CD8+ T cells in a melanoma model (15). Though some studies mentioned the expression difference of SIGLEC family members between normal and tumor tissue, functions of tumor derived SIGLEC gene expression on tumor growth and progression were rarely investigated. Our results showed all SIGLEC family genes expressed by tumors were survival (OS and PFS) related in various cancer types, and mutation frequencies or expression levels of them differed according to origin or tissue types of tumor, which needs further experiments to undermine the detailed mechanisms in different cancer types. Since SIGLEC genes expressed on immune cells can stimulate or inhibit immune cell function, we examined the correlation between tumor expressed SIGLEC genes and immune infiltration score, as well as immune regulators in tumor microenvironment. Enrichment of SIGLEC family highly correlated genes was also performed. It seemed tumor-expressed SIGLEC genes also were highly correlated with immune microenvironment of tumor, and they may regulate immune infiltration by influencing chemokine axis. SIGLEC family genes were correlated with macrophage, neutrophil, and dendritic cell infiltration in broad cancer types, and in specific tumors, correlation with B cell, CD4+ T cell, and CD8+ T cell infiltration levels were high. Former studies showed expression of SIGLEC genes on immune cells were positively correlated with immune checkpoints, such as PD1, and in our study, tumor expressed SIGLEC genes were also positively correlated with various immune stimulators and inhibitors (8, 36, 38, 42). Also, the recent study about SIGLEC15 additionally demonstrated the similar structure of SIGLEC15 and PDL1, which makes us wonder whether SIGLEC family genes expressed on tumor cells may directly influence immune cell infiltration and function in tumor microenvironment by binding to potential targets on immune cells, since SIGLEC15 has a relatively conservative structure among them (15, 43–45). Those results shed new light on immune blockade therapy to improve patients’ prognosis by neutralizing SIGLEC receptors on tumor cells or immune cells (12, 15, 46, 47).

There are several limitations of our study. First, the analysis was performed by using data from online databases, which needs further experiments for validation. Second, the immune infiltration status of different cancer types was calculated by computational methods. Though calculation was performed by multiple algorithms and correlation analysis was performed with tumor purification adjusted, the sequencing data may contain information from other cell sources, which requires tissue sample confirmation.

## Conclusion

Our computational analysis showed SIGLEC family genes expressed by tumor cells in different cancer types were related to tumor formation and patients’ survival, and they could regulate tumor immune microenvironment by influencing chemokine axis. Targeting tumor derived SIGLEC genes may benefit patients’ survival by both interfering with tumor malignant behaviors and improving tumor immune microenvironment.

## Data Availability Statement

Publicly available datasets were analyzed in this study. This data can be found here: https://www.cancer.gov/about-nci/organization/ccg/research/structural-genomics/tcga https://www.cbioportal.org http://timer.cistrome.org http://gepia.cancer-pku.cn http://kmplot.com/private/http://ualcan.path.uab.edu https://string-db.org https://david.ncifcrf.gov.

## Ethics Statement

Ethical review and approval was not required for the study on human participants in accordance with the local legislation and institutional requirements. Written informed consent for participation was not required for this study in accordance with the national legislation and the institutional requirements.

## Author Contributions

ZC, HL, and QY designed the investigation, and ZC, MY, QM, and ZY helped to collect data in databases. ZC, MY, LG, BIZ and SL wrote the draft of the paper, and WZ, BOZ, JY, YSX, and YFX helped to revise and make adaptations. All authors contributed to the article and approved the submitted version.

## Funding

This study was funded by National Nature Science Foundation of China (81871924 to QY, 81572844 to YFX).

## Conflict of Interest

The authors declare that the research was conducted in the absence of any commercial or financial relationships that could be construed as a potential conflict of interest.

## References

[1] Varki A Angata T Siglecs–the major subfamily of I-type lectins. Glycobiology (2006) 16:1r–27r. 10.1093/glycob/cwj008 16014749

[2] Crocker PR Redelinghuys P Siglecs as positive and negative regulators of the immune system. Biochem Soc Trans (2008) 36:1467–71. 10.1042/BST0361467 19021577

[3] Macauley MS Crocker PR Paulson JC Siglec-mediated regulation of immune cell function in disease. Nat Rev Immunol (2014) 14:653–66. 10.1038/nri3737 PMC4191907 25234143

[4] Bordon Y Inflammation: Live long and prosper with Siglecs. Nat Rev Immunol (2015) 15:266–7. 10.1038/nri3851 25882243

[5] Fraschilla I Pillai S Viewing Siglecs through the lens of tumor immunology. Immunol Rev (2017) 276:178–91. 10.1111/imr.12526 PMC5860639 28258691

[6] Lübbers J Rodríguez E van Kooyk Y Modulation of Immune Tolerance via Siglec-Sialic Acid Interactions. Front Immunol (2018) 9:2807. 10.3389/fimmu.2018.02807 30581432 PMC6293876

[7] Jandus C Boligan KF Chijioke O Liu H Dahlhaus M Démoulins T Interactions between Siglec-7/9 receptors and ligands influence NK cell-dependent tumor immunosurveillance. J Clin Invest (2014) 124:1810–20. 10.1172/JCI65899 PMC3973073 24569453

[8] Matsumoto T Takahashi N Kojima T Yoshioka Y Ishikawa J Furukawa K Soluble Siglec-9 suppresses arthritis in a collagen-induced arthritis mouse model and inhibits M1 activation of RAW264.7 macrophages. Arthritis Res Ther (2016) 18:133. 10.1186/s13075-016-1035-9 27267914 PMC4897938

[9] Pearce OM Läubli H Sialic acids in cancer biology and immunity. Glycobiology (2016) 26:111–28. 10.1093/glycob/cwv097 26518624

[10] Bärenwaldt A Läubli H The sialoglycan-Siglec glyco-immune checkpoint - a target for improving innate and adaptive anti-cancer immunity. Expert Opin Ther Targets (2019) 23:839–53. 10.1080/14728222.2019.1667977 31524529

[11] Reily C Stewart TJ Renfrow MB Novak J Glycosylation in health and disease. Nat Rev Nephrol (2019) 15:346–66. 10.1038/s41581-019-0129-4 PMC6590709 30858582

[12] van de Wall S Santegoets KCM van Houtum EJH Büll C Adema GJ Sialoglycans and Siglecs Can Shape the Tumor Immune Microenvironment. Trends Immunol (2020) 41:274–85. 10.1016/j.it.2020.02.001 32139317

[13] Cao G Xiao Z Yin Z Normalization cancer immunotherapy: blocking Siglec-15! Signal Transduct Target Ther (2019) 4:10–0. 10.1038/s41392-019-0045-x PMC6473001 31016034

[14] Ren X Immunosuppressive checkpoint Siglec-15: a vital new piece of the cancer immunotherapy jigsaw puzzle. Cancer Biol Med (2019) 16:205–10. 10.20892/j.issn.2095-3941.2018.0141 PMC6713637 31516742

[15] Wang J Sun J Liu LN Flies DB Nie X Toki M Siglec-15 as an immune suppressor and potential target for normalization cancer immunotherapy. Nat Med (2019) 25:656–66. 10.1038/s41591-019-0374-x PMC7175920 30833750

[16] Gao J Aksoy BA Dogrusoz U Dresdner G Gross B Sumer SO et al: Integrative analysis of complex cancer genomics and clinical profiles using the cBioPortal. Sci Signaling (2013) 6:pl1. 10.1126/scisignal.2004088 PMC4160307 23550210

[17] Tomczak K Czerwinska P Wiznerowicz M The Cancer Genome Atlas (TCGA): an immeasurable source of knowledge. Contemp Oncol (Poznan Poland) (2015) 19:A68–77. 10.5114/wo.2014.47136 PMC4322527 25691825

[18] Chandrashekar DS Bashel B Balasubramanya SAH Creighton CJ Ponce-Rodriguez I Chakravarthi B UALCAN: A Portal for Facilitating Tumor Subgroup Gene Expression and Survival Analyses. Neoplasia (New York NY) (2017) 19:649–58. 10.1016/j.neo.2017.05.002 PMC5516091 28732212

[19] Nagy Á Lánczky A Menyhárt O Győrffy B Validation of miRNA prognostic power in hepatocellular carcinoma using expression data of independent datasets. Sci Rep (2018) 8:9227. 10.1038/s41598-018-27521-y 29907753 PMC6003936

[20] Human genomics The Genotype-Tissue Expression (GTEx) pilot analysis: multitissue gene regulation in humans. Sci (New York NY) (2015) 348:648–60. 10.1126/science.1262110 PMC4547484 25954001

[21] Tang Z Li C Kang B Gao G Li C Zhang Z GEPIA: a web server for cancer and normal gene expression profiling and interactive analyses. Nucleic Acids Res (2017) 45:W98–w102. 10.1093/nar/gkx247 28407145 PMC5570223

[22] Li B Severson E Pignon JC Zhao H Li T Novak J Comprehensive analyses of tumor immunity: implications for cancer immunotherapy. Genome Biol (2016) 17:174. 10.1186/s13059-016-1028-7 27549193 PMC4993001

[23] Li T Fan J Wang B Traugh N Chen Q Liu JS TIMER: A Web Server for Comprehensive Analysis of Tumor-Infiltrating Immune Cells. Cancer Res (2017) 77:e108–10. 10.1158/0008-5472.Can-17-0307 PMC6042652 29092952

[24] Szklarczyk D Franceschini A Wyder S Forslund K Heller D Huerta-Cepas J STRING v10: protein-protein interaction networks, integrated over the tree of life. Nucleic Acids Res (2015) 43:D447–452. 10.1093/nar/gku1003 PMC4383874 25352553

[25] Shannon P Markiel A Ozier O Baliga NS Wang JT Ramage D Cytoscape: a software environment for integrated models of biomolecular interaction networks. Genome Res (2003) 13:2498–504. 10.1101/gr.1239303 PMC403769 14597658

[26] Chin CH Chen SH Wu HH Ho CW Ko MT Lin CY cytoHubba: identifying hub objects and sub-networks from complex interactome. BMC Syst Biol (2014) 8 Suppl 4:S11. 10.1186/1752-0509-8-S4-S11 25521941 PMC4290687

[27] Huang da W Sherman BT Lempicki RA Systematic and integrative analysis of large gene lists using DAVID bioinformatics resources. Nat Protoc (2009) 4:44–57. 10.1038/nprot.2008.211 19131956

[28] Kanehisa M Sato Y Furumichi M Morishima K Tanabe M New approach for understanding genome variations in KEGG. Nucleic Acids Res (2019) 47:D590–5. 10.1093/nar/gky962 PMC6324070 30321428

[29] R Core Team R: A language and environment for statistical computing. Vienna, Austria: R Foundation for Statistical Computing (2019). Available at: https://www.R-project.org/.

[30] RStudio Team RStudio: Integrated Development for R. Boston, MA: RStudio, Inc. (2015). Available at: http://www.rstudio.com/.

[31] Kolde R pheatmap: Pretty Heatmaps. R package version 1.0.12. (2019). Available at: https://CRAN.R-project.org/package=pheatmap.

[32] Wickham H ggplot2: Elegant Graphics for Data Analysis. Springer-Verlag New York (2016). ISBN 978-3-319-24277-4.

[33] Alexa A Rahnenfuhrer J topGO: Enrichment Analysis for Gene Ontology. R package version 2.36.0. (2019).

[34] Yu G Wang LG Han Y He QY clusterProfiler: an R package for comparing biological themes among gene clusters. Omics J Integr Biol (2012) 16:284–7. 10.1089/omi.2011.0118 PMC3339379 22455463

[35] Boligan KF Mesa C Fernandez LE and von Gunten S: Cancer intelligence acquired (CIA): tumor glycosylation and sialylation codes dismantling antitumor defense. Cell Mol Life Sci CMLS (2015) 72:1231–48. 10.1007/s00018-014-1799-5 PMC11113383 25487607

[36] Beatson R Tajadura-Ortega V Achkova D Picco G Tsourouktsoglou TD Klausing S The mucin MUC1 modulates the tumor immunological microenvironment through engagement of the lectin Siglec-9. Nat Immunol (2016) 17:1273–81. 10.1038/ni.3552 PMC5257269 27595232

[37] Huang PJ Low PY Wang I Hsu SD Angata T Soluble Siglec-14 glycan-recognition protein is generated by alternative splicing and suppresses myeloid inflammatory responses. J Biol Chem (2018) 293:19645–58. 10.1074/jbc.RA118.005676 PMC6314137 30377253

[38] Stanczak MA Siddiqui SS Trefny MP Thommen DS Boligan KF von Gunten S Self-associated molecular patterns mediate cancer immune evasion by engaging Siglecs on T cells. J Clin Invest (2018) 128:4912–23. 10.1172/jci120612 PMC6205408 30130255

[39] Yu Y Blokhuis BRJ Diks MAP Keshavarzian A Garssen J Redegeld FA Functional Inhibitory Siglec-6 Is Upregulated in Human Colorectal Cancer-Associated Mast Cells. Front Immunol (2018) 9:2138. 10.3389/fimmu.2018.02138 30294327 PMC6159741

[40] Barkal AA Brewer RE Markovic M Kowarsky M Barkal SA Zaro BW CD24 signalling through macrophage Siglec-10 is a target for cancer immunotherapy. Nature (2019) 572:392–6. 10.1038/s41586-019-1456-0 PMC6697206 31367043

[41] Santegoets KCM Gielen PR Büll C Schulte BM Kers-Rebel ED Küsters B Expression profiling of immune inhibitory Siglecs and their ligands in patients with glioma. Cancer Immunol Immunother: CII (2019) 68:937–49. 10.1007/s00262-019-02332-w PMC6529385 30953118

[42] Takamiya R Ohtsubo K Takamatsu S Taniguchi N Angata T The interaction between Siglec-15 and tumor-associated sialyl-Tn antigen enhances TGF-beta secretion from monocytes/macrophages through the DAP12-Syk pathway. Glycobiology (2013) 23:178–87. 10.1093/glycob/cws139 23035012

[43] Magesh S Ando H Tsubata T Ishida H Kiso M High-affinity ligands of Siglec receptors and their therapeutic potentials. Curr Med Chem (2011) 18:3537–50. 10.2174/092986711796642580 21756229

[44] Chang L Chen YJ Fan CY Tang CJ Chen YH Low PY Identification of Siglec Ligands Using a Proximity Labeling Method. J Proteome Res (2017) 16:3929–41. 10.1021/acs.jproteome.7b00625 28899088

[45] Bornhofft KF Goldammer T Rebl A Galuska SP Siglecs: A journey through the evolution of sialic acid-binding immunoglobulin-type lectins. Dev Comp Immunol (2018) 86:219–31. 10.1016/j.dci.2018.05.008 29751010

[46] Pfeifer M Zheng B Erdmann T Koeppen H McCord R Grau M Anti-CD22 and anti-CD79B antibody drug conjugates are active in different molecular diffuse large B-cell lymphoma subtypes. Leukemia (2015) 29:1578–86. 10.1038/leu.2015.48 25708834

[47] Adams OJ Stanczak MA von Gunten S Läubli H Targeting sialic acid-Siglec interactions to reverse immune suppression in cancer. Glycobiology (2018) 28:640–7. 10.1093/glycob/cwx108 29309569

